# An Experimental Study on the Shear Behaviors of Polymer-Sand Composite Materials after Immersion

**DOI:** 10.3390/polym10080924

**Published:** 2018-08-18

**Authors:** Jin Liu, Yuxia Bai, Ding Li, Qiongya Wang, Wei Qian, Ying Wang, Debi Prasanna Kanungo, Jihong Wei

**Affiliations:** 1 School of Earth Sciences and Engineering, Hohai University, Nanjing 210098, China; byxhhu@163.com (Y.B.); geofaris617@foxmail.com (D.L.); hhu_wqy@163.com (Q.W.); wei.geoserve@gmail.com (W.Q.); hhuwy1@163.com (Y.W.); weijhhhu@163.com (J.W.); 2 Council of Scientific and Industrial Research (CSIR)-Central Building Research Institute (CBRI), Roorkee 247667, India; debi.kanungo@gmail.com

**Keywords:** polymers, sand, composite materials, shear behavior, immersion

## Abstract

Sand mixed with geotextile/fiber/cement/lime or non-traditional chemical additives to form composite materials is recognized as an effective method for improving the sand properties. In this work, the variation in properties of composite materials after immersion is reported which has rarely appeared in the literature. The focus of this study is to evaluate the shear behaviors of polymer-sand composite material after immersion with direct shear tests. Several factors which may influence the shear behaviors after immersion are analyzed. The results demonstrate that this composite material still has good shear behaviors after immersion when compared to the purely sand material. The shear behaviors are improved with an increment in the curing time, polymer content and sand dry density while there is a decrease in the shear behaviors with increasing immersion time. The interaction between sand particles and the polymer are analyzed with Scanning Electron Microscope (SEM). The polymer membranes are formed by polymer enwrapping and connected sand particles to build an elastic and viscous structure in the sand that increases the interlocking forces between sand particles and decreases the void ratio of this material. The membranes are softened in water resulting in a decrease in the shear strength. Moreover, other factors affect the shear behaviors by improving the completeness and stability of this structure.

## 1. Introduction

As a natural material, sand is widely used in subgrade constructions, foundation work, and slope engineering as a buffer/backfill material with the advantages of being widely distributed, easy to draw and low-cost materials. However, the inherent characteristics of sandy soil with a loose structure, poor adhesion, and less clay content easily results in various geologic disasters, e.g., the slope surface easily tends to form gullies with water erosion and results in soil and water losses; saturated sand is prone to liquefy under dynamic loading, thus resulting in foundation failures. Erosion and bank collapses induced by the action of ship waves and river erosion usually occur in river banks.

Therefore, sand reinforcement is a hot topic in geotechnical engineering, and sand mixed with other reinforced materials to form a kind of composite materials is internationally recognized as an effective method for improving the sand’s properties [1,2]. In earlier times, people mixed sand with geomembrane/geotextile or cement/lime/fly ash to improve the physical and engineering behaviors of the soil to achieve a specific level of performance [3,4,5,6,7]. With in-depth research, fibers attracted the interested attention of scholars and researchers with several strengths [8,9,10,11], e.g., it is easily mixed randomly and evenly with soil, it avoids forming some potential weak planes with great strength, and it has gained plenty of important achievements [12,13,14,15]. The influence of fiber types including synthetic fiber, plant fiber, fiber sizes (length, diameter), and fiber contents (i.e., the weight ratio of fibers to soil) on the resistance of liquefaction, strength behaviors, and mechanical mechanism are researched in depth. On the other hand, the brittle behaviors of cement/lime treated soil, which is prone to cracking and may influence the stability of structures, play negative effects on the local environment [16,17,18,19,20]. Therefore, non-traditional chemical additives including liquid polymer, resins, acids, silicates, and ions are also used as a cost-effective and flexible option [21,22,23,24]. Liu et al. [25] illustrated that the presence of the organic polymers could effectively enhance the strength behaviors of the treated sand. Rezaeimalek et al. [26] discussed the influence of the curing method and mix design on the performance of stabilized sand with Methylene Diphenyl Diisocyanate. Ajalloeian et al. [27] investigated the variation in the improvement of sandy soil mixed with polyvinyl acetate in different dry densities. In addition, rheology is an important parameter to composite materials [28,29].

All the mentioned research achievements illustrate that these methods can usefully improve the sand behaviors. There are many studies on increasing strength, reducing permeability, improving the resistance to liquefaction, and weathering action [30,31,32,33,34]. However, very limited studies are carried out to evaluate the behaviors of composite materials after immersion. Moreover, the variation in properties of composite materials after immersion is crucial for roadways, embankments, levees, and water channel constructions [26].

In this study, sand is mixed with a polymer to form a composite material and the shear behaviors of the polymer-sand composite material after immersion are studied by methods of laboratory direct shear tests. Based on the tests results and comparison of the SEM photomicrographs before and after immersion, the interaction mechanism of the composite materials is analyzed in-depth. The research results and associated discussions provide the interaction mechanism of the composite materials for researchers and practicing engineers.

## 2. Materials and Methods

### 2.1. Sand and Polymer Stabilizer

The sand used in this study is collected from Nanjing, eastern China. The physical behavior of the sand is obtained by a series of laboratory tests and the results are shown in Table 1.

A polymer stabilizer, a light-yellow liquid, was used in this study. It contains plenty of macromolecular long chains, including abundant functional groups (–NCO) and hydrophilic groups. The polymer stabilizer is mainly prepared by the reaction of poly-oxypropylene diol (PPG, Jining Hongming Chemical Reagent Co. Ltd., Jining, China), poly-oxyethylene glycol (PEG, Shanghai Ika Biotechnology Co. Ltd., Shanghai, China), and an excessive dose of Toluene diisocyanate (TDI, Nantong Runfeng Petrochemical Co. Ltd., Nantong, China) [25,34]. The structural formula of the polymer is given as Formula (1), and the properties are shown in Table 2.

(1) $$O=C=N\;{\left[R_{1}-\mathrm{NH}-\mathrm{CO}-O-R_{2}-O-\mathrm{CO}-\mathrm{NH}\right]}_{n}R_{1}-N=C=O$$

where R_1_ = 
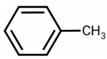
, R_2_ = PEG and/or PPG.

### 2.2. Polymer-Sand Composite Material Preparation

**Figure 1:**
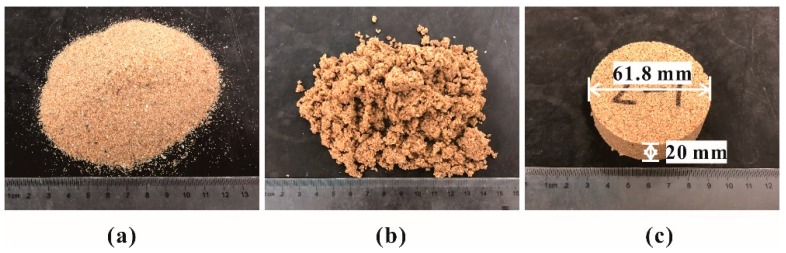
(**a**) The prepared sand; (**b**) prepared polymer-sand composite material; (**c**) prepared specimen.

The sand is mixed with the polymer at different weight ratios of the polymer to the soil (i.e., *W*_r_ = (*W*_p_/*W*_s_) × 100% = 0, 1%, 2%, 3%, 4%, 5%, Test Number T_13_–T_17_). The water content is maintained at 10% due to the sand-water characteristics. The following process obtains the polymer-sand composite material. Firstly, the prepared polymer and distilled water were added together to the container and stirred to get an evenly viscous solution. Then this solution was put into the prepared sand (Figure 1a) (i.e., *ρ* = 1.4, 1.5, 1.6 g/cm^3^, Test Number T_18_–T_20_) and mixed to obtain uniform polymer-sand mixture (Figure 1b). Secondly, the prepared mixture was placed into the cylinder molds with a diameter of 61.8 mm and a height of 20 mm. The static pressure was applied to the cylinder molds by a jack for several minutes. After that, the specific specimens (Figure 1c) were air cured at room temperature (20 ± 2 °C) for different times (i.e., *T*_c_ = 1, 6, 24, 48, 72 h, Test Number T_1_–T_5_). Finally, the cured specimen was placed into the transparent container filled with water. It was immersed for different times (i.e., *T*_i_ = 1, 6, 24, 48, 72, 168, 216 h, Test Number T_6_–T_12_). Thus, the polymer-sand specimens are obtained for further testing.

### 2.3. Direct Shear Test

The shear properties of the polymer-sand composite material after immersion are studied by the direct shear tests. They are carried out with ZJ typed strain control direct shear apparatus [25]. The shear stress-displacement curve is pictured separately under a normal stress (*σ*) of 100, 200, 300, 400 kPa at a shear rate of 2.4 mm/min. The shear strength (*τ*) is defined as the peak value or the value of shear stress corresponding to a 4 mm displacement of shear curves. The shear strength parameters including cohesion (*c*) and internal friction angle (*φ*) are calculated by Coulomb’s law of shear strength, as shown in Formula (2):

(2) $$\tau_{n}=\sigma_{n}\times \tan\varphi +c$$

where *τ_n_* (kPa) is the shear strength; *σ_n_* (kPa) is the normal stress; *φ* (°) is the internal friction angle; and *c* (kPa) is the cohesion. Cohesion (*c*) and the internal friction angle (*φ*) reflect the strength characteristics of the material itself and only change with the variety of the material.

## 3. Results

### 3.1. The SEM Studies and Analysis

The variation in the microstructure of the polymer-sand composite material was studied by scanning electron microscopy (SEM, SU3500, Hitachi, Chiyoda-ku, Tokyo, Japan) at an acceleration voltage of 5 kV and a beam current of 100 μA. The SEM specimen was prepared in the following manner. Firstly, the polymer-sand composite material specimen (2 mm × 2 mm × 1 mm, length × width × height) was dried in an oven at around 90 °C for 8 h. Secondly, the dried specimen was fixed on the object stage by double-sided adhesive tape and then a conductive silver paste was used to connect the sample surface and the object stage. After the conductive silver paste dried, the specimen surface was sputtered with gold at a voltage of 700 V for a minute by the ion sputter (E-1010, Hitachi, Chiyoda-ku, Tokyo, Japan). 

**Figure 2:**
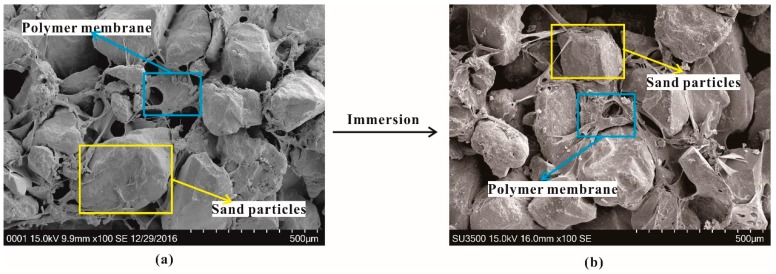
The SEM photomicrograph of the polymer-sand composite material for different periods: (**a**) Before immersion; (**b**) After immersion.

A photomicrograph from Scanning Electron Microscopy (SEM) of polymer-sand composite material before immersion was presented in Figure 2a. As seen, the polymer membrane formed by the reaction with the polymer and water, as shown in Formulas (3) and (4), builds physicochemical bonds between macromolecular long chains and sand particles. The –NCO groups of the polymer readily reacted with the –OH groups of water as shown in Formula (2); then the resulted product continued to react with the polymer to form macromolecular chains as shown in Formula (3). Thereby, it built bonds between the macromolecular long chains and sand particles. Moreover, the membrane is insoluble in water.

(3) $$O=C=N-R-N=C=O+2H_{2}O\to \mathrm{HO}-\mathrm{CO}-\mathrm{NH}-R-\mathrm{NH}-\mathrm{CO}-\mathrm{OH}\to H_{2}N-R-{\mathrm{NH}}_{2}+2{\mathrm{CO}}_{2}.$$

(4) $$\left(n+1\right)H_{2}N-R-{\mathrm{NH}}_{2}+\mathrm{nO}=C=N-R-N=C=O\to H_{2}N\;{\left[R-\mathrm{NH}-\mathrm{CO}-\mathrm{NH}\right]}_{2n}\;R-{\mathrm{NH}}_{2}$$

**Figure 3:**
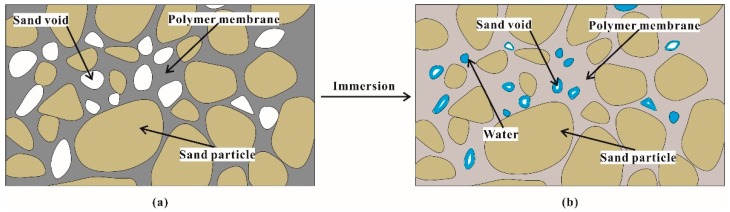
The schematic diagram of the polymer-sand composite material for different periods: (**a**) Before immersion; (**b**) After immersion.

With these bonds, the membranes enwrap and connect sand particles with each other to form an elastic and viscous structure in the polymer-sand mixture. The formed structure would increase the bonding and interlinking forces, and decrease the void ratio of the polymer-sand mixture. Therefore, the characteristics of the polymer-sand composite material have been improved. The SEM image of the polymer-sand mixture immersed in water for 9 days is presented in Figure 2b. It can be observed that the polymer membrane still holds a complete and stable structure in the polymer-sand mixture. While the specimens are immersed in water, the membranes softened and swelled (Figure 3), resulting in a decrease of the strength characteristic.

### 3.2. Direct Shear Test Results

**Figure 4:**
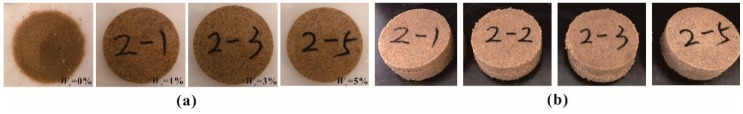
The shape of the polymer-sand composite material for different periods: (**a**) After immersion for 48 h; (**b**) After carrying out the direct shear test.

The trials of the laboratory direct shear tests were conducted on the polymer-sand composite material after immersion. Several factors that will influence the shear behaviors are investigated in detail, including the curing time (*T*_c_), polymer content (*W*_r_), sand dry density (*ρ*), and immersion time (*T*_i_). The direct shear tests results are summarized in Table 3. Figure 4a presents the shape of the specimens with different polymer contents (i.e., *W*_r_ = 0%, 1%, 3%, 5%) after immersion in water for 48 h. As shown, the specimen with 0% polymer disintegrates totally while those specimens mixed with the polymer remain intact without any disintegration or destruction behaviors. Such behavior is induced by the appearance of the polymer membranes, which improves the adhesion between the sand particles. It is noted that the zero value approach of the adhesion of sand was considered as a reference because it is difficult to carry out a direct shear test on the disintegrated specimen. It can be known from Figure 4b that the specimens’ body largely remains intact after carrying out direct shear tests. There is just a small amount of shear displacement in the middle section of the specimens. The detail analyses with variation in the shear properties of the polymer-sand composite material after immersion are given in the following section.

#### 3.2.1. The Effect of Curing Time on Shear Behaviors

**Figure 5:**
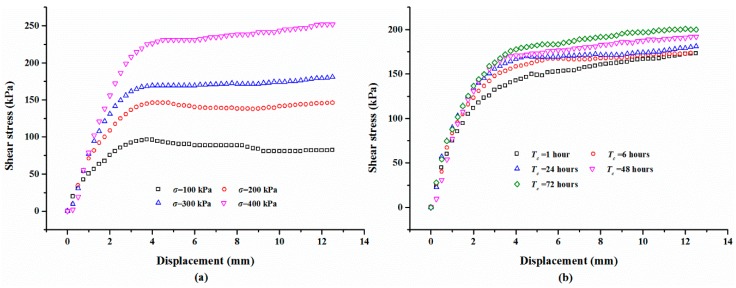
The typical shear curves:(**a**) With different normal stress *σ*; (**b**) With different curing times *T*_c_.

**Figure 6:**
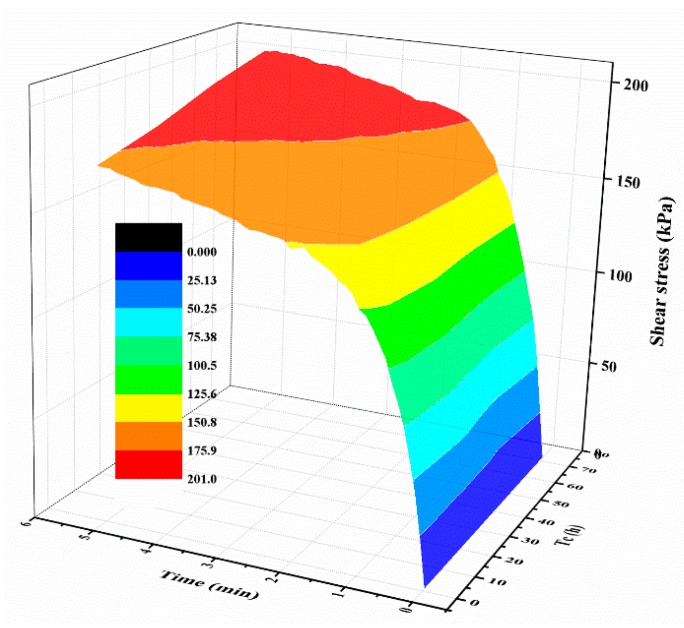
The relationships between shear stress, test time, and *T*_c_.

Figure 5a shows the typical shear curves of the specimens with different normal stresses. The mixture specimens were prepared with 2% polymer at 1.4 g/cm^3^ dry density, 48 h of air curing, and 48 h of immersion in water. As expected, the shear stress increases monotonically with the increase of the shear displacement before the shear strength is reached. After that, the shear stress maintains at a stable value with increasing displacement. Additionally, the shear stress of the mixture specimen with a large normal stress has a bigger value than the ones with small normal stress. The representative shear curves of the mixture specimen with a normal stress of 300 kPa cured for different times is shown in Figure 5b, and it is observed from the figure that, prior to reaching the shear strength, the slopes of the curves were not significantly affected by the curing time. Additionally, with the increasing displacement, the curing time plays an active effect on the shear stress behavior. Figure 6 displays the relationship between shear stress, test time, and curing time. As shown obviously in the figure, the shear stress increases rapidly with an increment in the test time, and increases slowly to maintain a stable value while the shear strength is reached. Figure 6 also shows that the curing time plays an active role on the shear stress when the shear strength is reached.

**Figure 7:**
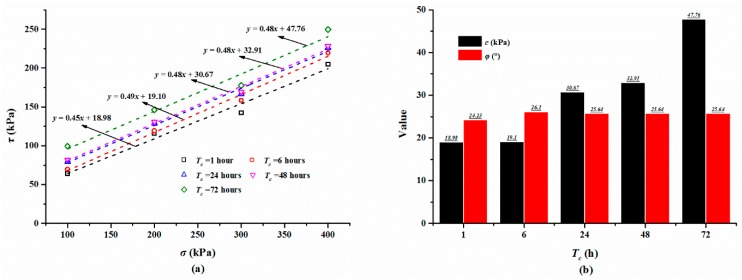
(**a**) The relationship between shear strength *τ* and normal stress *σ*; (**b**) The relationship between the shear strength parameters (*c* and *φ*) and curing time *T*_c_.

The shear strengths of the specimens cured for different times are summarized in Table 3 and Figure 7a displays the relationship between shear strength and normal stress. As shown in Figure 7a, the shear strength of the specimen with a large normal stress is higher than that with a small normal stress and increases slightly with an increase in the curing time. For the specimen cured for 48 h, the shear strength increased from 81.73 to 228.39 kPa, while the normal stress increased from 100 to 400 kPa (Table 3). Additionally, for the mixture specimen with a normal stress of 300 kPa, cured for an hour, 6, 24, 48, and 72 h, the shear strength is 142.30, 158.30, 166.34, 169.22, 177.56 kPa, respectively (Table 3). Based on these shear strength, Coulomb’s law of shear strength was used to compute the cohesion and internal friction angle of the composite material (Figure 7a). The calculated cohesion and internal friction angle are presented in Table 3 and Figure 7b. As expected, the cohesion shows an increasing trend with increments in the curing time. The cohesion values of the specimens cured for 6, 24, 48, and 72 h are 19.10, 30.67, 32.91, and 47.76 kPa, respectively. The cohesion is increased by 0.12, 11.69, 13.93, and 27.78 kPa compared to that of 18.98 kPa with one hour of curing time. Figure 6b also shows that the internal friction angle varies gently with the increase in the curing time and fluctuates at a value of 25°.

#### 3.2.2. The Effect of Immersion Time on Shear Behaviors

**Figure 8:**
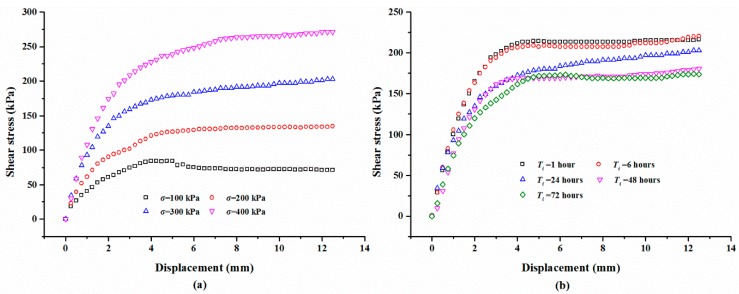
The presentative shear stress-displacement curves: (**a**) With different normal stress *σ*; (**b**) With different immersion time *T*_i_.

**Figure 9:**
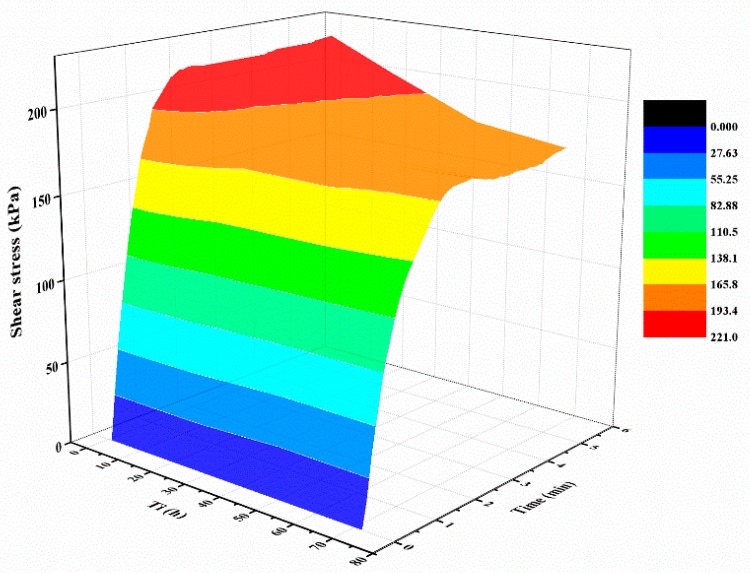
The relationships between shear stress, test time, and *T*_i_.

The polymer membrane formed by the polymer stabilizer would be softened while immersed in water, which may change the behaviors of the composite material. The effect of the immersion time on the shear properties is studied by carrying out direct shear tests. Figure 8a shows the typical shear curves of the specimen compacted with a 2% polymer at a dry density of 1.4 g/cm^3^ cured for 48 h and immersed in water for 72 h. It can be observed that the shear stress of all the mixture specimens follow a relatively gentle gradient with increasing displacement, and then remain stable with an increment in the displacement. Figure 8a also displays that the normal stress plays an active effect on the shear behavior of the mixture specimen. Figure 8b presents the shear curves of the specimens tested on a normal stress of 300 kPa. It can be seen that the shear behavior of the specimen is reduced with an increase in the immersion time. The variation in the shear stress of the specimens tested with a normal stress of 300 kPa with the test time and immersion time is shown in Figure 9. As shown, the shear stress presents a clear variation trend with increasing test time, increasing at first to the peak value and then keeping stable around this value. It also can be seen from Figure 9 that the shear stress decreases with an increment in the immersion time when the test time is over two minutes.

**Figure 10:**
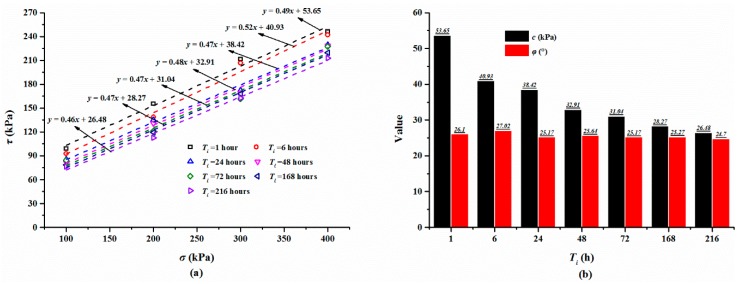
(**a**) The variation of the shear strength *τ* and normal stress *σ*; (**b**) The variation of the shear strength parameters (*c* and *φ*) and immersion time *T*_i_.

The shear strengths of the mixture specimens immersed in water for different times are given in Table 3 and Figure 10a. They all obviously show that the shear stress increases with an increase in the normal stress, and decreases with an increase in the immersion time. While the normal stress increases from 100 to 400 kPa with an increment of 100 kPa, the shear strength keeps stable at about 80, 120, 160, and 220 kPa, respectively. For the specimen immersed in water for different times (i.e., *T*_i_ = one hour, 6, 24, 48, 72, 168 and 216 h) and tested with a normal stress *σ* = 400 kPa, the shear strength is 246.14, 242.04, 229.62, 228.39, 227.52, 219.61, and 212.94 kPa, respectively. The calculated shear strength parameters of the specimens are shown in Table 3 and Figure 10b. As shown, the cohesion changes with a decreasing trend with the increasing immersion time, and the cohesion keeps stable at a value of about 30 kPa while the specimen is immersed in water for more than 9 days. It also can be observed from Figure 10b that the friction angle varies gently with the change in immersion time, and is maintained steady at 25° when the immersion time exceeds 24 h.

#### 3.2.3. The Effect of Polymer Content on Shear Behaviors

**Figure 11:**
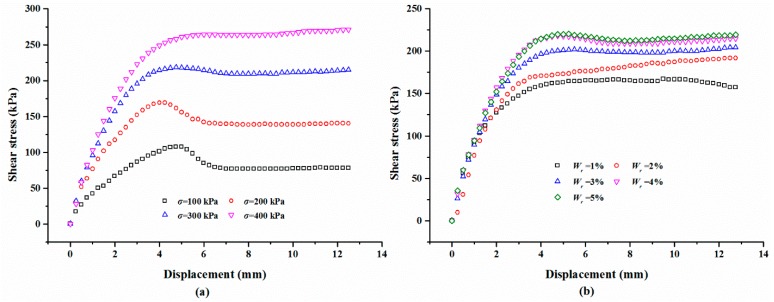
The typical shear curves: (**a**) With different normal stress *σ*; (**b**) With different polymer content *W*_r_.

**Figure 12:**
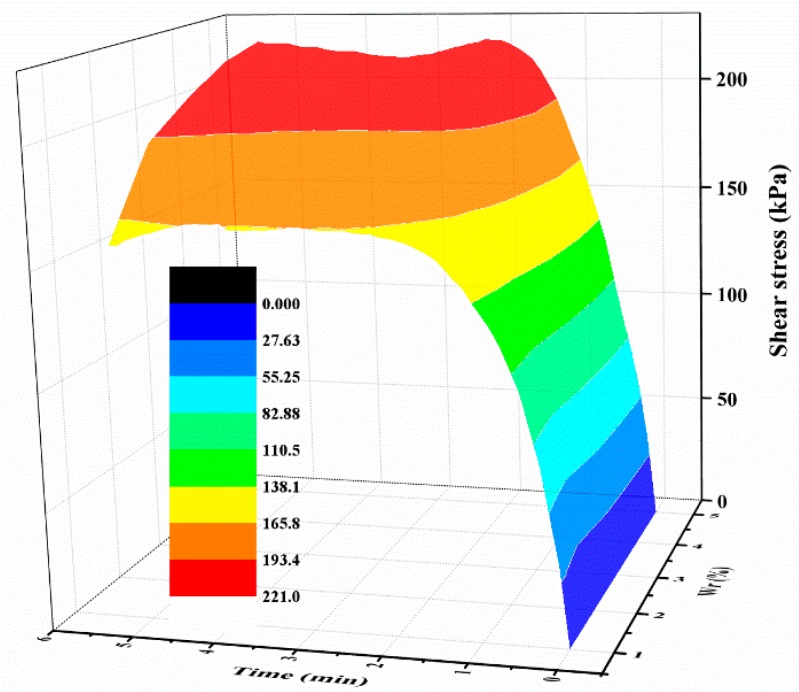
The relationships between shear stress, test time, and *W*_r_.

The polymer content may play a vital role in the stability of the polymer membrane and it significantly influences the strength of the polymer-sand composite material. Thereby, the polymer content in the prepared specimen varies from 1% to 5% with an increment of 1%. The typical shear stress-displacement curves of the mixture specimen with various normal stresses are given in Figure 11a. It can be seen that the shear stress of the mixture with a normal stress of 100 and 200 kPa increases first to its peak shear strength, and then decreases gently, suggesting that the shear failure occurs at the center of the edge of the specimen. Finally, the shear stress maintains a stable value with increasing shear. Figure 11a also shows that the shear stress of the material tested with a larger than normal stress value increases first to the shear strength value and then remains stable at that value. Figure 11b displays the shear curves of the mixture specimens prepared with different polymer contents tested on a normal stress of 300 kPa. It is observed that polymer content has a vital effect on the shear behavior of the specimen, especially when the shear stress reaches the shear strength. Figure 12 is the relationships between the shear stress, test time, and polymer content. As seen in Figure 12, with the test time increasing, the shear stress shows a similar trend as those of Figure 6 and Figure 9. When the test time is over about two minutes, the shear stress keeps stable with the increasing test time, but increases with the increase in the polymer content.

**Figure 13:**
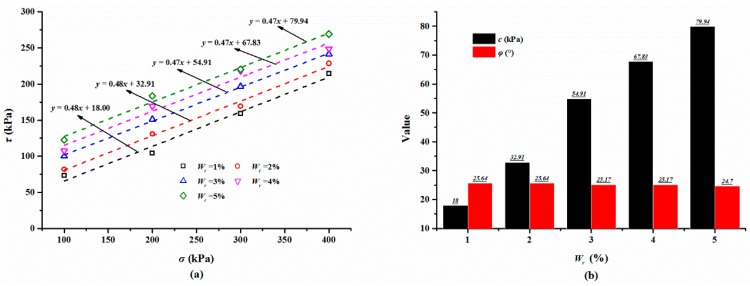
(**a**) The linear relationship between the shear stress *τ* and normal stress *σ*; (**b**) The relationship between the shear strength parameters (*c* and *φ*) and the polymer content *W*_r_.

The shear strength obtained from the shear curves is summarized in Table 3 and Figure 13a presents the relationship between the shear strength and normal stress. As seen, the shear strength shows an increasing linear trend with an increase in the normal stress, and it increases with the increase in the polymer content. For the mixture specimen compacted with a 3% polymer tested at a normal stress increasing from 100 to 400 kPa, the shear strength varies from 99.99 to 241.20 kPa. The results of the shear strength parameters are given in Figure 13b. As expected, the cohesion of the mixture increases greatly with the polymer content varying from 1% to 5%. However, the internal friction angle changes lightly with the varying polymer contents. While the polymer content of the mixture increases from 1% to 5% with an increment of 1%, the cohesion of mixture is 18.00, 32.91, 54.91, 67.83, and 79.94 kPa, respectively and internal friction angle is 25.64°, 25.64°, 25.17°, 25.17° and 24.70°, respectively, being stable around 25°.

#### 3.2.4. Effect of Sand Dry Density on Shear Behaviors

**Figure 14:**
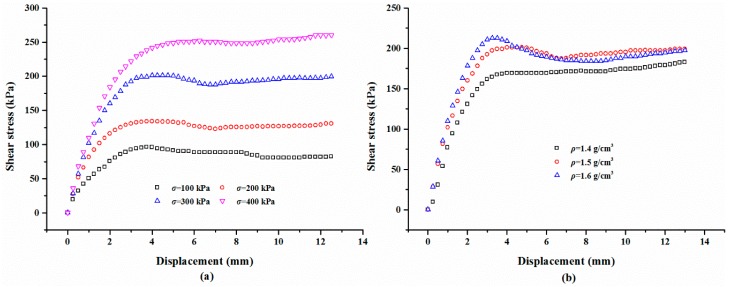
The presentative shear stress-displacement curves: (**a**) With different normal stress *σ*; (**b**) With different dry density *ρ*.

**Figure 15:**
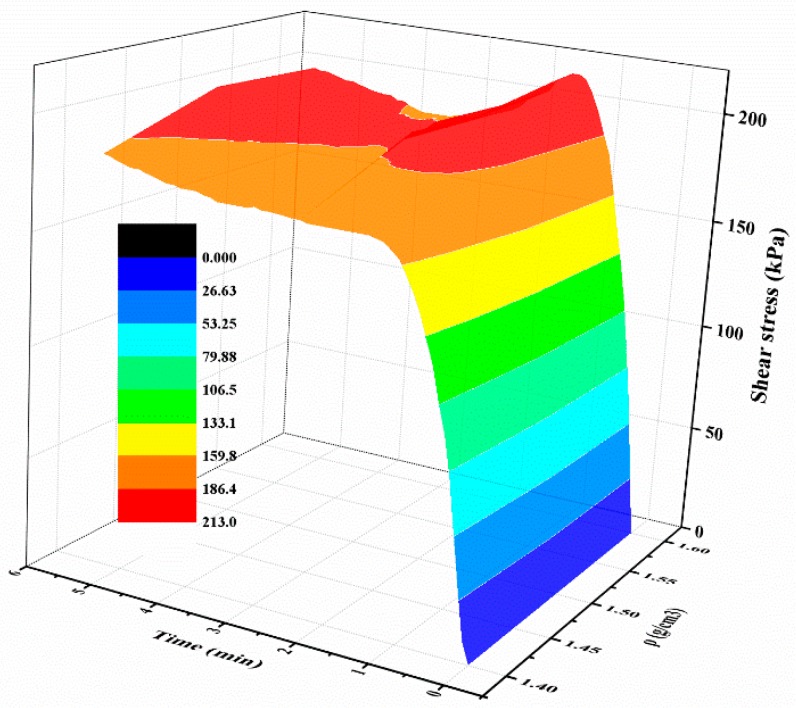
The relationship between shear stress, test time, and *ρ*.

For the polymer-sand composite material, limited studies have been carried out to investigate the influence of dry density on the mechanical property. This aspect was studied by performing direct shear tests on the composite material with different dry densities. The representative shear curves of the mixture specimens prepared with a 2% polymer at 1.4 g/cm^3^, cured for 48 h, and immersed in water for 48 h, are tested with different normal stresses and shown in Figure 14a. As seen, with any normal stress, the shear stress of all the specimens increases first to the shear strength value, and then keeps stable around the peak value. It also can be observed that the shear stress is significantly influenced by normal stress. Figure 14b presents the shear curves of the specimens at different dry densities tested on a normal stress of 300 kPa. As shown, the dry density did not significantly affect the overall shape of the shear curves of the specimens and is in agreement with the result presented in Figure 14a. However, the dry density increases the shear strength of the specimen. Figure 15 displays the variation in the shear stress of specimens tested with a normal stress of 300 kPa and with the test time and dry density. As expected, the shear stress of the specimens in dense conditions increases rapidly to the peak value and then decreases to a relatively stable value. However, the specimens in a loose condition maintain stability when the peak strength is reached. Figure 15 also shows that the shear stress increases with an increment in the dry density when the test time is over about two minutes.

**Figure 16:**
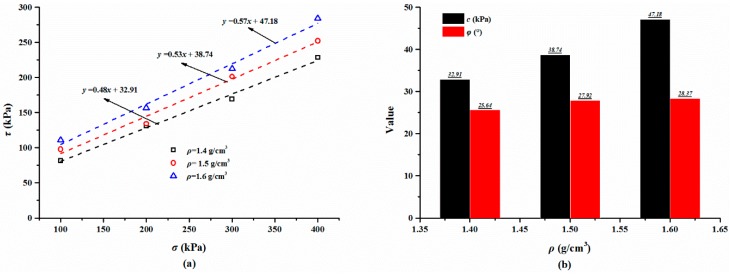
(**a**) The linear relationship between the shear strength *τ* and normal stress *σ*; (**b**) The relationship between the shear strength parameters (*c* and *φ*) and the dry density *ρ*.

The variation in the shear strength with different dry densities for the specimens tested with different normal stresses can be observed in Figure 16a. The shear strength increases linearly with an increase in the dry density regardless of the normal stress. For instance, for specimens tested with a normal stress of 300 kPa, the shear strength is 169.22 kPa at a dry density of 1.4 g/cm^3^ and it increases to 212.35 kPa at a dry density of 1.6 g/cm^3^, respectively (Table 3). Another feature observed in Figure 16a is that the dry density affects the linear relationship between the shear strength and normal stress. The results of the shear strength parameters are presented in Figure 16b. As expected, the shear strength parameters increase with an increment in dry density. For the specimens prepared at dry densities of 1.4, 1.5, and 1.6 g/cm^3^, the cohesion values are 32.91, 38.74, and 47.18 kPa the and internal friction angles are 25.64°, 27.92°, 28.37°, respectively.

## 4. Discussion

**Figure 17:**
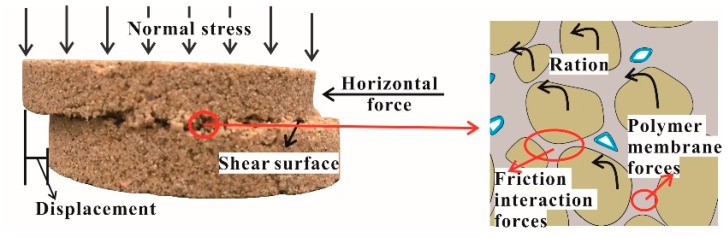
The schematic diagram of the specimen during the shear processing.

It is generally true that the action of the friction and cohesion determines the mechanical properties of the soil [35]. The specimens become denser when bearing different normal stresses, thus resulting in a great improvement in the frictional interaction forces, and this is the reason why the shear behaviors strengthen with increments in the normal stress. When the specimen bears a horizontal force, with increasing test time, the sand particles are rotated, displaced, and rearranged, and the polymer membrane and frictional interaction forces between sand particles cause them to resist the movement of the sand particles (Figure 17). Sand is a kind of problematic soil with poor cohesion, a loose structure, and less clay content, the adhesion depends on the membrane strength and the condition of the membrane enwrapping sand particles (i.e., the completeness and stability of the elastic and viscous structure formed with the polymer membrane in the sand). On the other hand, the frictional interaction forces are influenced by the surface roughness of the soil particles. The membranes absorbed on the surface of the soil particles soften and swell, reducing the surface roughness. Additionally, the lubrication action induced by water leads to a slight decrease in the frictional interaction forces between the soil particles.

As mentioned earlier, while the prepared polymer solution is mixed with sand, the polymer membranes formed with the reaction of the polymer and water on the surface of the sand particles or sand voids change the frictional interaction forces between the sand particles by forming a three-dimensional bridge between the macromolecular long chains and the detached sand particles. Once these are formed in the polymer-sand composite material, they increase the inter-particle cohesive forces of the mixture, although it depends on *T*_c_, *T*_i_, *W*_p_, and *ρ*.

The polymer and water need enough time to react to form polymer membranes, and increasing the reaction time strengthens the material strength behaviors by enhancing the connection forces between the sand particles [31,34]. On the other hand, with an increment in the curing time of the specimen, a greater strength, more elasticity, and a softer membrane structure in the mixture can be achieved due to the fact that the liquid fractions of the specimens evaporate [19]. The test results demonstrate that the specimen still has the load-bearing capacity although it is immersed in water for 48 h and the curing time plays an active role on the shear behaviors of the specimens after immersion.

As shown before, the shear properties of the mixture after immersion in water showed a decreasing trend with the increasing immersion time and the dominant change in the mechanism of the strength properties is based on the action of the water. The specimen soaked with water for a long time makes an increment in the water content of the specimens, resulting in a decrease of the load-bearing capacity of the specimen [36,37]. On the other hand, the polymer membranes are insoluble, softened in the water with infiltration, and have negative effects on the shear performance of the specimen. Additionally, with increasing immersion time, while the water content varies and the membrane softens to reach a saturation state, the shear strength characteristics remain stable.

Considering that the polymer membranes enwrap and connect the detached sand particles, the strength of the polymer-sand composite material depends on the stability of the three-dimensional structure formed by the membranes in the sand particles. The specimens prepared at a higher polymer content have a more stable three-dimensional bridge with a large adhesion surface contact through the soil particles and stronger membranes and, therefore, they improve the strength performances of the specimens.

The dry density dependant on the sand density condition influences the completeness and stability of the three-dimensional structure that affects the strength properties of the specimens. For the specimen with a dense structure, the sand particles are tightly touched and arranged, resulting in the incompletely wrapping of the sand particles by the membranes because of a small adhesion surface contact and the mostly fill up sand voids. With a decrease in the dry density, the membranes easily enwrap the sand particles but build a relatively weakened connection between the macromolecular long chains and the sand particles because of the loose structure of the specimens. Additionally, dry density plays a significant positive role in the interaction of the particles [27]. Thus, the shear behaviors of the specimens improve with an increment in the dry density.

## 5. Conclusions

This study carried out trials of direct shear tests on a polymer-sand composite material after immersion to determine the influence of curing time *T*_c_, immersion time *T*_i_, polymer content *W*_r_, and dry density *ρ* on the shear behavior. Additionally, the interaction between the polymer and sand is analyzed by these test results and the SEM study. The results obtained in this study can be summarized as follows:

(1) The polymer-sand composite material still has good shear behaviors after immersion when compared to the only sand material. An increment in the curing time, polymer content, and dry density displays improvements in the shear properties, including the shear curves, shear strength, and shear strength parameters. The variation of the curing time and polymer content leads to a great change in the cohesion, while weakly affecting the friction angle, which fluctuating at around 25°. Both cohesion and the friction angle increase with an increment in the dry density, which varies from 32.91 to 47.18 kPa and 25.64° to 28.37° while the dry density increases from 1.4 to 1.6 g/cm^3^. However, the increase in the immersion time reduces the shear behaviors of this composite material after immersion. Similar to the curing time and polymer content, the immersion time significantly influences the cohesion and lightly affects the friction angle.
(2) The shear behaviors of the polymer-sand composite material mainly depend on the integrity and stability of the elastic and viscous structure formed by the polymer membrane. When the specimen is immersed in water, the water content of the specimens increases. In the meantime, the membranes are softened, and therefore, the shear strength decreases with an increment in the immersion time. However, the curing time and polymer content both play positive effects on the integrity and stability of the three-dimensional bridge by increasing the membrane strength and adhesion contact of the sand particles. Unlike the curing time and polymer content, the sand dry density affects the membranes’ integrity through the tightness of the soil particle arrangement and determines the frictional action of soils. Thus, the shear strength of this material improves with an increase in the dry density.

## Figures and Tables

**Table 1:** The basic behaviors of the used sand.

**Weight Percentage Content (%)**	**Size Distribution Range of Sand Particle (mm)**						
	2–1		1–0.5		0.5–0.25	0.25–0.1	0.1–0.075
	0.2 ^1^		17.1		48.9	31.7	2.1
**Physical Parameters**	Dry Density *ρ* (g/cm^3^)		Void Ratio *e*		Specific Gravity *G_s_*	Uniformity Coefficient *C_u_*	Gradation Coefficient *C_c_*
	*ρ_max_* = 1.69	*ρ_min_* = 1.36	*e_max_* = 0.97	*e_min_* = 0.59	2.65	2.27	1.13

^1^ The weight of sand particles, which ranged from 1 to 2 mm accounts for 0.2% of the total sand. Others are similar to it.

**Table 2:** The properties of the polymer stabilizer.

PH	Specific Gravity *G_s_*	Viscosity (mPa·s)	Solid Content (%)	Coagulation Time (s)
7	1.15	650–800	88	60–1600

**Table 3:** The direct shear test results of the specimens after immersion.

Test Number	Polymer Content (%)	Dry Density (g/cm^3^)	Curing Time (hour)	Immersion Time (hour)	Shear Strength (kPa)				Shear Strength Parameters	
					*σ* = 100 kPa	*σ* = 200 kPa	*σ* = 300 kPa	*σ* = 400 kPa	*c* (kPa)	*φ* (°)
T_1_	2	1.4	1	48	63.69	115.38	142.30	204.80	18.98	24.23
T_2_	2	1.4	6	48	69.23	119.23	158.30	219.48	19.10	26.10
T_3_	2	1.4	24	48	79.24	128.84	166.34	225.95	30.67	25.64
T_4_	2	1.4	48	48	81.73	130.76	169.22	228.39	32.91	25.64
T_5_	2	1.4	72	48	99.53	146.15	177.56	249.70	47.76	25.64
T_6_	2	1.4	48	1	99.03	155.38	211.53	246.14	53.65	26.10
T_7_	2	1.4	48	6	92.73	138.46	206.72	242.04	40.93	27.02
T_8_	2	1.4	48	24	85.57	135.30	173.07	229.62	38.42	25.17
T_9_	2	1.4	48	48	81.73	130.76	169.22	228.39	32.91	25.64
T_10_	2	1.4	48	72	84.23	121.15	162.54	227.52	31.04	25.17
T_11_	2	1.4	48	168	78.46	119.23	167.83	219.61	28.27	25.27
T_12_	2	1.4	48	216	76.61	112.69	162.51	212.94	26.48	24.70
T_13_	1	1.4	48	48	73.07	104.23	159.06	214.37	18.00	25.64
T_14_	2	1.4	48	48	81.73	130.76	169.22	228.39	32.91	25.64
T_15_	3	1.4	48	48	99.99	151.02	196.62	241.20	54.91	25.17
T_16_	4	1.4	48	48	107.69	169.22	218.11	248.95	67.83	25.17
T_17_	5	1.4	48	48	122.69	183.65	220.54	269.14	79.94	24.70
T_18_	2	1.4	48	48	81.73	130.76	169.22	228.39	32.91	25.64
T_19_	2	1.5	48	48	97.61	134.22	201.17	251.99	38.74	27.92
T_20_	2	1.6	48	48	110.76	156.72	212.35	283.88	47.18	28.37
